# Low dose DTIC is effective and safe in pretreated patients with well differentiated neuroendocrine tumors

**DOI:** 10.1186/s12885-016-2642-1

**Published:** 2016-08-18

**Authors:** Daniela Mueller, Sebastian Krug, Moushumee Majumder, Anja Rinke, Thomas Matthias Gress

**Affiliations:** 1 Department of Gastroenterology, University Hospital Marburg, Baldinger Strasse, D35043 Marburg, Germany; 2 Department of Gastroenterology, University of Halle, Ernst-Grube-Straße 40, D 06120 Halle, Germany

**Keywords:** Dacarbazine, Neuroendocrine tumor, Chemotherapy, Objective response, Prognostic markers

## Abstract

**Background:**

Streptozocin (STZ) based chemotherapy is recommended for patients with metastatic pancreatic neuroendocrine tumors (pNET). Temozolomide as mono- or combination therapy has been suggested to be a promising alternative. However, the treatment is costly and not approved for the treatment of pNETs. Dacarbazine (DTIC) shares the active metabolite with temozolomide and is broadly available at a low cost. The aim of this study was a retrospective evaluation of the efficacy and tolerability of a lower dose DTIC-regimen in patients with progressive advanced NETs.

**Methods:**

We retrospectively analyzed 75 patients with NETs predominantly of pancreatic origin treated at our center between 1998 and 2013. 650 mg/m^2^ of DTIC were administered intravenously over 60 min every 4 weeks. Morphological response was assessed according to RECIST1.1 criteria. The median progression free survival (PFS) was calculated using Kaplan-Meier and Cox regression methods, respectively. Univariate analyses of possible prognostic markers were performed.

**Results:**

The objective response rate (ORR) was 27 % for the entire cohort and 32 % in 50 pNET patients, respectively. Stable disease (SD) was documented in 29 patients (39 %). Median PFS (mPFS) in patients receiving DTIC was 7 months (3.9–10; 95 % confidence interval). Radiological and biochemical response were the only significant prognostic markers for longer PFS in univariate analysis. Treatment was well tolerated. Nausea was the most common side effect (31 %), only one case (1.3 %) of grade 3 toxicity (vomiting) occurred.

**Conclusion:**

Low dose DTIC chemotherapy is an effective and well-tolerated treatment option in patients with progressive well differentiated neuroendocrine neoplasms, especially of pancreatic origin.

**Electronic supplementary material:**

The online version of this article (doi:10.1186/s12885-016-2642-1) contains supplementary material, which is available to authorized users.

## Background

With the advent of novel molecular targeted treatments such as everolimus and sunitinib the therapeutic armamentarium in metastatic neuroendocrine tumors (NET) has broadened. Chemotherapy is still considered first line treatment in specific patient subgroups such as patients with neuroendocrine carcinoma where platinum-based chemotherapy is recommended [1]. The European Neuroendocrine Tumor Society (ENETS) suggests using a streptozocin-based chemotherapy as first line treatment in metastatic pancreatic neuroendocrine tumors (pNET) G1 and G2 with a high tumor burden or tumor-related local symptoms [2] and as second line treatment in progressive pNET. In recent years some small studies reported promising results for the alkylating agent temozolomide as single- or combination therapy. In a retrospective study first-line treatment of 30 patients with pancreatic neuroendocrine tumors (pNET) with temozolomide and capecitabine resulted in an impressive response rate of 70 % and a 2 year survival rate of 92 % [3]. Dacarbazine is an alkylating agent sharing the active metabolite metozolomide with temozolomide. Different regimens of dacarbazine have been used for more than three decades. Early reports included clinical and morphological responses in patients with glucagonoma syndrome [4, 5]. The largest monotherapy study so far comprised 50 patients with progressive pNET treated with 850 mg/m2 dacarbazine every 4 weeks. The response rate of this Eastern Cooperative Oncology Group study was 33 %. Most responders had not received prior chemotherapies [6]. This protocol was associated with relevant toxicities including two deaths, 13 % grade 3 vomiting and 10 % grade 4 hematotoxicity. In another large randomized trial of the Eastern Cooperative Oncology Group dacarbazine as second line treatment in patients with carcinoid tumors resulted in a response rate of only 8.2 % [7].

In this study we retrospectively investigated the efficacy and safety of a modified, less dose intense dacarbazine treatment schedule comprising single intravenous applications of 650 mg/m^2^ DTIC every 4 weeks in a cohort of 75 mostly pretreated patients with well differentiated neuroendocrine neoplasms consecutively treated at our institution.

## Methods

### Patients

We retrospectively evaluated 75 consecutive patients with histologically confirmed well differentiated neuroendocrine neoplasms who were treated at our hospital with dacarbazine between 1998 and 2013. All patients had measurable disease according to the Response Evaluation Criteria in Solid Tumors (RECIST 1.1). A total of 40 male and 35 female patients with a median age of 56 years (range 28–80) were treated. Patient characteristics are summarized in Table 1.

**Table 1:** Summary of patient characteristics

Characteristic	Number	Percent
Age (years)	median: 56	range: 27–78
< 60	49	65.3
> 60	26	34.7
Sex		
Male	40	53
Female	35	47
Localization of primary		
Pancreas	50	66.6
Midgut	11	14.6
Bronchus	6	8
Stomach	1	1.3
Hindgut	2	2.6
Thymus	1	1.3
Unknown	4	5.3
Tumor grading		
G1	27	36
G2	34	45
Unspecified	14	19
Prior treatment		
1 prior treatment	24	32
> 1 prior treatment	43	57
No pretreatment	4	5.3
No data available	4	5.3
Metastases		
No metastases (locally advanced)	2	2.6
Liver only	33	44
Liver plus additional Extrahepatic metastases (Bone, lung, spleen)	40	53

For all but four patients dacarbazine represented at least the second line of treatment, median number of pretreatments was two (0–5). Thus the cohort is representative for heavily pretreated patients (details of the pretreatments are given as Additional file 1: Table S1).

### Treatment and evaluation

DTIC treatment was initiated after documented tumor progression. 650 mg/m2 of DTIC were administered intravenously over 60 min every 4 weeks. All patients received granisetron as antiemetic premedication.

Patients were restaged every 3 months using CT or MRI-scans and by measuring serum chromogranin A (CgA) levels. Biochemical response was defined as a decrease of CgA of at least 30 %. Morphological response was assessed according to RECIST1.1 criteria. Side effects were collected from the medical files and classified according to the common toxicity grading system (Common Terminology Criteria for Adverse Events Version 4.0).

### Statistical analysis

The median progression free survival (PFS) was calculated using Kaplan-Meier and Cox regression methods, respectively. Univariate analyses were performed. All statistical calculations were performed using SPSS (IBM SPSS Statistics). Differences were considered statistically significant when the P value was less than 0.05. The primary endpoint was the objective response rate (ORR). Secondary endpoints included progression free survival (time from first dose of chemotherapy until documentation of tumor progression or death), duration of response and toxicity.

## Results

### Efficacy

A median of eight courses of DTIC (range 3–46) were administered. A biochemical response was observed in 19 of 39 patients (49 %) with elevated plasma CgA before dacarbazine therapy. Median PFS (mPFS) in patients receiving DTIC was 7 months (3.9–10; 95 % confidence interval). A partial remission (PR) could be documented in 20/75 patients (27 %). Responding patients had well differentiated NETs of pancreatic (*n* = 16), intestinal (*n* = 1), bronchial (*n* = 2) and gastric (*n* = 1) origin. PR lasted a median of 24 months (95 % confidence interval: 17.4–30.5). Stable disease was observed in 29 patients (39 %):17 of pancreatic, one of colonic, five of intestinal, three of bronchial, two of unknown primary and one of thymic origin. Stable disease lasted a median of 13 months (95 % CI: 11.3–14.8). Disease progression occurred in 26 patients (35 %).

**Fig. 1:**
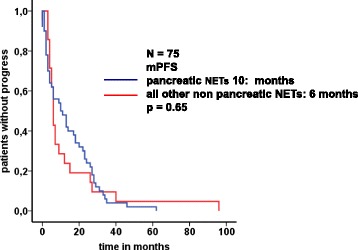
Kaplan-Meier survival curves for progression free survival of pancreatic versus non-pancreatic NETs; mPFS: median progression free survival. Overall progression free survival tended to be longer for pancreatic (10 months) than for non pancreatic NETs (6 months) without statistical significance (*p* = 0.65)

The separate analysis of NENs of pancreatic origin demonstrated a partial remission in 32 % (*n* = 16/50) with a median progression free survival of 27 months (95 % CI: 23.1–30.9). Stable disease was found in 17 patients (34 %) who had a median progression free survival of 18 months (95 % CI: 13.7–22.3). Progressive disease occurred in 17 patients (32 %) with pancreatic NEN. As shown in Fig. 1, overall progression free survival was 10 months for pancreatic and 6 months for all non pancreatic NETs, this difference, however, was not statistically significant (*p* = 0.65).

A univariate analysis of potential prognostic markers for PFS revealed that only radiologic and biochemical response to DTIC were significant prognostic markers. Patients with G1 tumors tended to have a longer PFS compared to patients with G2 tumors (*p* = 0.058). Differences in sex, age, functional activity, site of primary and number of pretreatments were not statistically significant (Table 2).

**Table 2:** Univariate analysis of possible prognostic factors for progression free survival

Variable	Hazard ratio (95%CI)	*P* value
Sex		
Female Male	1 1.2 (0.8–1.9)	0.46
Diagnosis		
FNA FA	1 0.9 (0.5–1.6)	0.64
Age start CTx		
< 60 ≥ 60	1 1.1 (0.7–1.7)	0.73
Grading		
G1 G2	1 1.7 (0.98–2.9)	0.058
Ki-67		
≤ 10 % > 10 %	1 1.1 (0.6–2.1)	0.76
Metastases		
Liver only Liver and others	1 1.0 (0.7–1.6)	0.91
Response (RECIST)		
No Yes	1 0.3 (0.18–0.56)	0.000*
Biochemical response		
No Yes	1 0.5 (0.3–0.9)	0.019*
Primary		
Non-pancreatic Pancreatic	1 0.8 (0.5–1.4)	0.51
Prior therapy		
No CTx CTx	1 0.8 (0.4–1.5)	0.41
Prior therapy		
SSA/IFN Others	1 0.9 (0.5–1.4)	0.61

*significant differences; *CI* confidence interval, *FNA* functional non active, *FA* functional active, *CTx* chemotherapy, *SSA* somatostatin analogue, *IFN* interferon

### Toxicity

Chemotherapy related adverse effects were documented in 43 patients of the entire study cohort (57.3 %) but were usually mild with only one case of grade 3 toxicity (vomiting, 1.3 %). The most common side effects were nausea (*n* = 23 (31 %)) and vomiting (*n* = 13 (17 %)). Transient elevations of liver enzymes were noted in 12 patients (15 %). Mild hematotoxicity was observed in eight patients (10.7 %). Other side effects were diarrhea (15 %), fatigue (5 %) and weight loss (3 %). Only two patients stopped DTIC due to intolerable side effects. Detailed information on chemotherapy related adverse effects is given in Table 3.

**Table 3:** Toxicity

Side effects	n	%	Grade 1	Grade 2	Grade 3
Hematologic					
Anemia	4	5	4	–	–
Leukopenia	6	8	5	1	–
Thrombocytopenia	2	3	–	2	–
Gastrointestinal					
Nausea	23	31	22	1	–
Vomiting	13	17	10	2	1
Diarrhea	11	15	7	4	–
Liver					
Elevated liver enzymes	12	15	11	1	–
Other					
Fatigue	4	5	4	–	–
Weight loss	2	3	2	–	–

## Discussion

Streptozocin based chemotherapies have been the mainstay of treatment of metastatic pNET since they were first described by Moertel in 1980 with response rates up to 69 % [8]. These report rates have been questioned by others [9] because of partial reliance on nonmorphologic response criteria including clinical and biochemical assessments. Nevertheless, the efficacy of streptozocin (STZ) based therapies has been confirmed by several groups [10–16] with documented response rates of 30–40 % according to radiologic criteria. Thus, STZ-based therapies have been recommended for the treatment of metastatic pNET by all international guidelines. However, disadvantages of the STZ based protocols include the need for a 5 days course and the risk of nephrotoxicity (9 % in the Moertel group) that could preclude potential second line therapies such as PRRT.

In this study, we report on the efficacy and tolerability of low dose dacarbazine in a large single center cohort. Dacarbazine (DTIC) is a well known agent that has safely and efficiently been used for the treatment of malignant melanomas and glioblastomas for many years. In NEN patients combination treatments of low doses of DTIC (200 or 250 mg/m^2^) with 5-FU and epirubicin resulted in response rates between 18 and 44 % [17–20]. In addition, some older studies used DTIC 250 mg/m^2^ in a 5 day treatment schedule [21–23], such as Altimari and coworkers who demonstrated response rates of 50 % in a small series of 14 patients.

Evidence for the efficacy of single dose DTIC monotherapy is limited. Bukowski and coworkers investigated the use of DTIC in a series of 56 patients with NENs of various primaries at a dose of 650 and 850 mg/m^2^ showing response rates of 20 % for the 850 mg/m^2^ dose and 13 % for the 650 mg/m^2^ dose [24]. Ramanathan and coworkers reported a response rate of 33 % in 50 patients with pancreatic NEN who received DTIC at a dose of 850 mg/m^2^. The response rate was higher in treatment naïve patients (50 %) compared to pretreated patients (14 %) [6]. Sun et al. observed a response rate of only 8 % with DTIC (250 mg/m^2^ d1-d5, every 4 weeks) after failure to STZ/5-FU or STZ/Doxorubicin chemotherapy in patients with advanced neuroendocrine tumors mainly of extrapancreatic origin [7]. The interpretation of the results needs some caution, since the cohorts show major differences. In all but one study NENs of all origins were included which explains some of the lower response rates, since chemotherapy is mostly effective in pNETs but not in midgut tumors, an observation as well confirmed in our study. Furthermore the studies differ in the number of patients that were pretreated, the number of pretreatments and other prognostic factors that could have influenced the treatment outcome like grading or tumor load. However, the majority of studies suggest that DTIC-based chemotherapy is active in well-differentiated NENs, though questions remain about the dosing schedule achieving the highest efficacy at an acceptable toxicity.

In accordance with these previously reported results we could clearly demonstrate in our retrospective study that DTIC chemotherapy is effective in 75 patients with well differentiated NEN, which represents the biggest series reported so far. 66 % of the patients had a benefit from DTIC chemotherapy documented by an overall partial remission (PR) in 27 % and disease stabilization (SD) in 39 % of the patients. As expected, efficacy varied depending on the primary site of the tumor. It has to be stressed that in our series all but four patients received DTIC after disease progression to at least one prior treatment which renders the response rate even more remarkable. Median PFS in pNEN was 10 months and thus comparable to the results of everolimus and sunitinib in progressive pNET patients. Remarkably, in patients with benefit we observed sustained response or stabilization periods, lasting a median of 27 months in patients with PR and 18 months for patients with SD. These results are comparable or only slightly inferior to those achieved with the established streptozocin based protocols.

Efficacy in small bowel NENs which are known to be less chemosensitive was low (PR 9 %), whereas four of six bronchus carcinoids benefited from DTIC therapy (PR 33 %, SD 33 %), suggesting that this regimen may also be effective in other foregut NENs. In the same way Ekeblad and coworkers included 13 bronchial carcinoids in their study using temozolomide and found a response rate of 31 % [25]. Since the number of patients with NEN of this origin was low in both series, further studies are needed to clarify the efficacy of temozolomide and dacarbazine for bronchial NEN.

In contrast to previous reports primarily with more dose intense DTIC schedules in our study DTIC demonstrated a good safety profile. While in our study side effects were common and were documented in 57 % of the patients, they were usually mild and transient. Only two patients stopped DTIC due to side effects. Dose adjustments were necessary in only three other cases. Notably, in contrast to former studies our patients did not experience grade 3 and 4 hematotoxicity. For instance, Ramanathan et al. reported 30 % grade 3 and 4 toxicities including 10 % grade 4 hematotoxicity [6] probably due to the use of a higher dose of DTIC of 850 mg/m^2^.

Temozolomide is closely related to dacarbazine and shares the same active metabolite. It is available in an oral formulation, crosses the blood–brain barrier and has recently been advocated as effective, well tolerated and convenient chemotherapy for patients with NEN. Temozolomide as a single therapeutic agent has been studied by Maire and coworkers in 21 mostly pretreated NEN patients [26]. They showed a relative low response rate of 5 %, but a disease stabilization in 81 %. The Uppsala group reported comparable results using temozolomide monotherapy in patients with advanced NEN (*n* = 36) achieving a response rate of 14 % and a stable disease rate of 53 % [25]. Although comparisons between these studies are always very limited due to the fact that patient characteristics differ significantly, in our study DTIC does not appear to be inferior to the results reported for temozolomide monotherapy.

In combination with capecitabine, temozolomide has shown a high response rate of 70 % in one small retrospective study of pancreatic NEN [3] suggesting synergistic effects between both drugs. However, all the patients in this study were chemotherapy naïve. Temozolomide in combination with capecitabine therefore seems to be a promising alternative to streptozocin based therapy of metastasized pancreatic NENs. A randomized prospective trial comparing both regimens is needed to determine which is the most effective regimen that should be used in first line treatment. Most temozolomide +/− capecitabine studies reported more severe side effects, in particular grade 3 hematologic toxicity (10–15 %) than observed in our trial. Authors have also reported a considerable incidence of opportunistic infections (10 %) related to this therapy which has rarely been reported in DTIC monotherapy and was not observed in our series [27]. Although DTIC has the disadvantage of an intravenous application route, patient discomfort is negligible, since treatment comprises a single infusion every 4 weeks in an outpatient setting. In direct comparison of the two regimens, the cost of the drug may be of relevance, since the price of a standard 5 day temozolomide course in Germany is approximately 14 times higher than the price of a single DTIC infusion. Since dacarbazine in our experience shows similar efficacy as temozolomide, we have just started to evaluate the efficacy and safety of combining dacarbazine with capecitabine.

Temozolomide has as well been used as second line chemotherapy in a small series of G3 NEN and demonstrated a response rate of 33 % [28]. Response rates were higher in tumors with Ki 67 < 60 % and in patients with positive somatostatin receptor imaging, indicating that these tumours had a higher degree of differentiation. Interestingly, adding capecitabine to temozolomide brought no additional benefit in this small series. In the same way, Olsen et al. found a response rate of 38 % in 25 patients with NECs using temozolomide alone [29]. Temozolomide may therefore be a therapeutic alternative in NEC G3 tumors with a Ki 67 index between 21 and 60 % which are not as aggressive as undifferentiated small cell G3 neuroendocrine cancers and often do not respond to standard cisplatin/etoposide therapy.

Apart from conventional chemotherapy the use of everolimus and sunitinib has provided novel and effective treatments for pNETs. However, the response rates achieved with these new agents overall are lower than with conventional chemotherapy and side effects can be considerable. The combination of temozolomide and everolimus has recently shown to be active in pNETs, with a response rate of 40 %, which is not superior to the results obtained with temozolomide plus capecitabine [30].

First promising results have as well been reported for the combination of temozolomide/capecitabine and peptide receptor radiotherapy (PRRT) in pNET with response rates of 82 % [31].

Thus, conventional chemotherapy with drugs such as dacarbazine, temozolomide and/or capecitabine shows strong activity in pNET and should be included in current treatment algorithms. They may also represent the backbone for trials of novel combination therapies.

## Conclusions

In summary, treatment with low dose (650 mg/m^2^) dacarbazine monotherapy demonstrated efficacy in progressive well differentiated NEN patients. Toxicity rates are considerably lower than those observed with high dose DTIC and combination treatments including streptozocin based regimens. Thus, low dose dacarbazine may be a therapeutic alternative for patients with well differentiated pancreatic NETs, in particular as second-line therapy. Future prospective randomized trials are necessary to further evaluate its role in patients with progressive well-differentiated NENs of pancreatic and lung origin.

## Abbreviations

5FU, 5 flourouracil; CgA, chromogranin A; CT, computed tomography; Ctx, chemotherapy; DTIC, dacarbazine; ENETS, European Neuroendocrine Tumour Society; FA, functional active; IFN, interferon; mPFS, median progression free survival; MRI, magnetic resonance imaging; NEC, neuroendocrine carcinoma; NEN, neuroendocrine neoplasia; NET, neuroendocrine tumor; PFS, progression free survival; pNET, pancreatic neuroendocrine tumor; PR, partial remission; PRRT, peptide receptor radiotherapy; SD, stable disease; SSA, somatostatin analogue; STZ, streptozocin

## Additional file

Additional file 1: Table S1. Summary of pretreatments of our patient cohort: 25 patients received one prior treatment, 33 patients received two prior treatments, six patients received three prior treatments, two patients four prior treatments and one patient five prior treatments before starting dacarbazine. (DOCX 18 kb)
