# Supplementary material for: Low dose DTIC is effective and safe in pretreated patients with well differentiated neuroendocrine tumors
Source: BMC Cancer. 2016 Aug 18;16:645. doi: 10.1186/s12885-016-2642-1 (PMC4989525; doi:10.1186/s12885-016-2642-1)
Supplement: Additional file 1: Table S1. — Summary of pretreatments of our patient cohort: 25 patients received one prior treatment, 33 patients received two prior treatments, six patients received three prior treatments, two patients four prior treatments and one patient five prior treatments before starting dacarbazine. (DOCX 18 kb) [file 12885_2016_2642_MOESM1_ESM.docx]

Additional file 1 Table S1: Summary of pretreatments.

33 patients received 2 prior treatments, 6 patients received 3 prior treatments, two patients 4 prior treatments and one patient 5 prior treatments before starting dacarbazine. FU: fluorouracil, doxo: doxorubicin; strepto: streptozocin, TACE: transarterial chemoembolisation; PRRT: peptide receptor radionuclide therapy, SIRT: selective internal radiotherapy; IFN: interferon

| **Type of pretreatment** | **No.** | **%** |
| --- | --- | --- |
| none | 4 | 5.6 |
| unknown | 4 | 5.6 |
| strepto/doxo or strepto/5FU | 19 | 25.3 |
| other chemotherapies | 6 | 8.0 |
| TACE | 14 | 19.7 |
| other liver directed treatments | 4 | 5.6 |
| PRRT | 2 | 2.8 |
| IFN | 30 | 42.3 |
| octreotide / lanreotide | 49 | 69 |
